# Does a Split-Week Gestational Age Model Provide Valuable Information on Neurodevelopmental Outcomes in Extremely Preterm Infants?

**DOI:** 10.3390/children8090731

**Published:** 2021-08-26

**Authors:** Elizabeth Asztalos, Alberto Nettel Aguirre, Leonora Hendson, Paige Church, Rudaina Banihani, Jessie van Dyk, Hussein Zein, Sumesh Thomas

**Affiliations:** 1 Sunnybrook Health Sciences Centre, Department of Paediatrics, University of Toronto, Toronto, ON M4N 3M5, Canada; paige.church@sunnybrook.ca (P.C.); rudainha.banihani@sunnybrook.ca (R.B.); 2 Departments of Paediatrics and Community Health Sciences, Cumming School of Medicine, University of Calgary, Calgary, AB T2N 4N1, Canada; alberto.nettel-aguirre@albertahealthservices.ca; 3 Department of Pediatrics, Cumming School of Medicine, University of Calgary, Calgary, AB T2N 4N1, Canada; leonora.hendson@albertahealthservices.ca; 4 St Joseph’s Health Centre, Department of Paediatrics, University of Toronto, Toronto, ON M6R 1B5, Canada; jvandyk@stjoestoronto.ca; 5 Foothills Medical Centre, University of Calgary, Calgary, AB T2N 2T9, Canada; hussein.zein@albertahealthservices.ca (H.Z.); sumesh.thomas@albertahealthservices.ca (S.T.)

**Keywords:** extreme preterm infant, neurodevelopmental outcomes, counseling

## Abstract

Our primary objective for this follow-up study was to compare the neurodevelopmental outcomes of a surviving cohort of infants using a split-week gestational model (early versus late) gestational age (GA) and the standard completed GA categorization. Neurodevelopmental outcomes using a split-week GA model defined as early (X, 0–3) and late (X, 4–6), with X being 23–26 weeks GA, were compared to outcomes using completed weeks GA. In total, 1012 infants were included in the study. Statistically significant differences were noted in outcomes between the early and late split of the gestational week at 23 weeks (early vs. late), with 13.3% vs. 54.5% for no neurodevelopmental impairment, and 53.3% vs. 22.7% for significant impairment (*p* = 0.034), respectively. There were no differences seen in the split week model for 24, 25, and 26 weeks. A trend towards improved neurodevelopmental outcomes was seen with each increasing gestation week. The split-week model did not provide additional information for pregnancies and infants between 24 and 26 weeks gestation. It did, however, provide information for counsel for infants at 23 weeks gestation, showing benefits in the late versus early half of the week.

## 1. Introduction

Over the last two decades, survival rates for infants born less than 27 weeks gestation have steadily improved. These improved survival rates have led to more active interventions for even more immature fetuses below 25 weeks gestation who may be at higher risk of serious neonatal morbidities, such as bronchopulmonary dysplasia/chronic lung disease, severe brain injury, and retinopathy of prematurity. Recent population studies evaluating neurodevelopmental outcomes in infants born at less than 25–26 weeks gestation focus on three main areas of disability: impairment of cognition, motor function, and neurosensory function. These findings relate to the objective assessment of cognitive function, a measure of vision and hearing impairment including the need for aids, and motor challenges primarily in the form of cerebral palsy and other categories of motor challenges relating to ambulation [1,2,3,4,5,6].

The current standards of counseling for families at risk of having an infant born at the limits of viability (≤26 weeks gestation) have typically utilized outcome statistics based on completed weeks of gestational maturity [7]. It is also known that the incidence of serious neonatal morbidity changes significantly from one week to the next and, in fact, decreases with increasing gestation [8]. Several studies also point out that infants born in the latter part of a gestational week have improved survival outcomes compared to those born in the early part of the week [9,10]. These studies did not examine the long-term consequences of this difference in their survivors. Long-term outcome information is likely to help parents faced with difficult decisions with the potential birth of a baby at the margins of viability, during neonatal care, and at the time of discharge from the hospital. Similarly, this information may be helpful to care providers in considering care options in consultation with informed families.

A previously conducted study including 1012 infants born between 23 and 26 weeks gestation at two similar perinatal centres showed statistically significant differences in the composite outcome of neonatal mortality or morbidity between the early and late split of each week at 24, 25, and 26 weeks of gestational maturity, suggesting that a delay in delivery of up to 72 h may lead to an improvement in survival for infants born at 24–26 weeks. The study used a split-week gestation age (GA) model, with an early (X, 0–3) versus late (X, 4–6) week split, where “X” was the gestation week, and 0–3 and 4–6 represented days and the first and latter part of the week, respectively [11]. It was interesting to note that this difference was not seen at 23 weeks gestation; however, the number of infants included in this study at 23 weeks gestation may have been too small to identify if a real difference existed at this gestation point using the split model approach. This finding contrasted that of Nguyen et al., which showed an increase in neonatal morbidity and mortality at the early versus late part of the week, even at 23 weeks gestation.

Both these studies, however, raise important questions about how the short-term outcome differences based on the early versus late part of gestational week maturity impact long-term outcomes for infants born at the extremes of viability. While information about the variance in short-term outcomes based on changes in gestational maturity by a factor of about 72 h is meaningful, understanding the longer-term consequences of these findings could provide clearer information to assist with counseling, both in the antenatal as well as neonatal settings.

The hypothesis in this study was that an early (X, 0–3) versus late (X, 4–6) week GA model may demonstrate differences in neurodevelopmental outcomes for infants born at the margins of viability in the same cohort where short-term outcome differences were demonstrated. Therefore, the primary objective for this follow-up study was to study the neurodevelopmental outcomes of the surviving cohort of 1012 infants born between 23 and 26 weeks gestation and discharged home, using a split-week gestational model (early versus late) gestational age (GA) in comparison to outcomes based on standard completed GA categorization.

## 2. Methods

This is the follow-up phase of the initial retrospective cohort study of the live-born infants at GA 23^0/7^ to 26^6/7^ weeks who were admitted to the neonatal intensive care units (NICUs) at the Foothills Medical Centre, Calgary, Alberta and Sunnybrook Health Sciences Centre, Toronto, Ontario between 1 January 2005 and 31 December 2014. Research Ethics Board approval was obtained for the study from Sunnybrook Health Sciences Centre (26 October 2016) and Foothills Medical Centre University of Calgary (5 November 2015). These infants were then seen in the neonatal follow-up programs of their respective institutions.

To summarize the elements of the initial phase of the project, both Foothills Medical Centre and Sunnybrook Health Sciences Centre are regional perinatal referral centres for high-risk pregnancies in Alberta and Ontario, respectively. Both sites were comparable in admission rates of infants <28 weeks gestation and had similar shared practices and approaches to the care of infants born between 23 and 26 weeks gestation over the study period. These included the use of antenatal corticosteroids for women at risk of a preterm birth ≤32 weeks gestation and the use of surfactant for respiratory distress. The use of a postnatal corticosteroid was similar and often limited to those infants at high risk of severe bronchopulmonary dysplasia and prolonged ventilatory support. Gestational maturity was assessed on antenatal ultrasound scans. Exclusion criteria included congenital anomalies, congenital infections, extremely growth restricted (less than the first percentile) [12], and infants who received palliative or comfort care measures only from delivery, as per parental wishes.

Following discharge home from the hospital, surviving infants were followed on a routine basis in their respective neonatal follow-up clinics. Between 18 and 24 months of age CA, the infants were assessed for the presence or absence of motor, visual, and hearing difficulties. Information on these difficulties was gathered during the 6-month period with the hopes that all information would become available by 2 years CA. In addition, where and when it was possible, the infants underwent a neurodevelopmental assessment, Bayley Scales of Infant and Toddler development–3rd edition (BSID-III) [13]. The BSID-III is a norm-referenced instrument to assess cognitive, language, and motor functions, as well as social-emotional and adaptive behaviors. The cognitive scale consists of items that are scaled and converted to a composite score. The language scale consists of two subtests, including receptive communication and expressive communication, whereas the motor scale entails fine motor and gross motor subtests. For the language and motor scales, the two subtests are scaled and combined to form the composite score of each of those domains. The composite scores for the three domains are age-standardized with a mean score of 100 and a standard deviation of 15.

Maternal and neonatal data were recorded from the initial study [11]. Recorded neurodevelopmental data included the presence and type of cerebral palsy, the Gross Motor Functional Classification System (GMFCS) score [14], the presence and severity of any hearing and vision impairment, and the completion of the BSID-III and its three composite scores (cognition, language, and motor). The GMFCS is a measure of functional ambulatory skills in the presence of cerebral palsy. The scores range from 1 to 5 with higher numbers reflecting a greater degree of impairment.

The primary outcome was significant neurodevelopmental impairment (SNI) and was a composite of at least one of the following: (i) BSID-III mean cognitive, language, or motor score <70 (2 standard deviations from norm mean of 100); (ii) the presence of cerebral palsy with a GMFCS of 3, 4, or 5; (iii) severe hearing impairment; or (iv) severe visual impairment. The secondary outcome was a mild neurodevelopmental impairment and was a composite of the following: (i) BSID-III mean cognitive, language, or motor score of 70–84 (1 standard deviation from norm mean of 100); (ii) the presence of cerebral palsy with a GMFCS of 1 or 2; (iii) a mild/moderate hearing impairment; or (iv) a mild/moderate visual impairment. Table 1 outlines the categorization of the neurodevelopmental outcome measures utilized in the study.

### Statistical Analyses

Because this was an exploratory analysis, we did not know of or set an effect size in the primary outcome based on early- versus late-week increments; thus, a power-based sample size was not calculated.

Descriptive statistics, means, and standard deviations (SD) or median and interquartile range (IQR) were used for numerical/continuous variables as appropriate (based on symmetry in the distribution of the data), while frequencies and proportions were used to summarize demographic data for categorical variables. As in the initial study, to test the hypothesis of a difference of early to late points within each week, tests of difference in proportions were performed within each nominal week. Since multiple pair-wise comparisons were being done, a Benjamini–Hochberg correction was used for multiple pair-wise comparisons. Adjustments were made for maternal education, as this was known to contribute to neurodevelopmental outcomes in the preterm population [15]. No additional adjustments were made for the analysis.

A level of significance of alpha = 0.05 was used in all statistical tests.

## 3. Results

**Figure 1:**
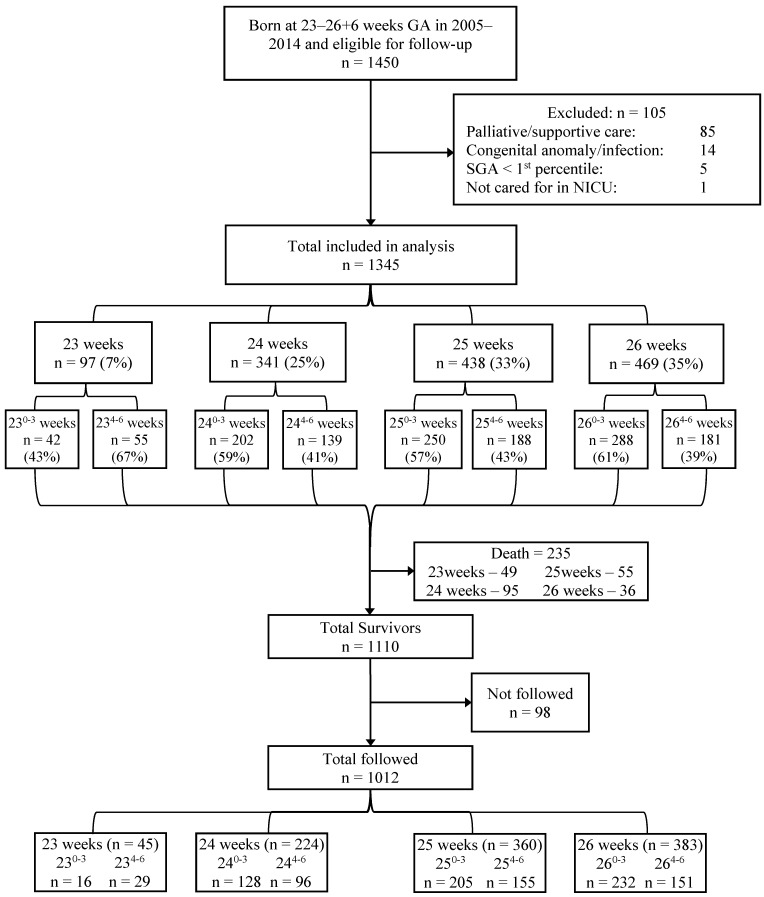
Flowchart of enrolled infants.

Figure 1 outlines the flow of infants included in the neonatal and neurodevelopmental phases of the principal project. Of the 1450 infants born and admitted to the NICU at both the Foothills Medical Centre and Sunnybrook Health Sciences Centre over the study period with a gestational age of 23–26 weeks, 1345 were eligible for inclusion in the neonatal phase; 235 infants died in the neonatal period, leaving 1110 eligible for inclusion in the neurodevelopmental phase.

For the neurodevelopmental phase, 1012 infants (91% of the eligible) were able to be followed and provided data up to the 18–24month assessment. We identified 45 infants in the 23-week GA group, 224 in the 24-week GA group, 360 in the 25-week GA group, and 383 in the 26-week GA group. Maternal and neonatal characteristics of those infants who survived are summarized in Table 2; the neurodevelopmental characteristics of those who provided data for the primary outcome are summarized in Table 3.

Table 4 outlines the primary outcome. A trend towards improved neurodevelopmental outcomes was seen with each increasing completed gestational week. Significant neurodevelopment impairment was noted in 35.1% in 23 completed weeks gestation, 22.4% in 24 completed weeks gestation, 13.8% in 25 completed weeks gestation, and 7.6% in 26 completed weeks gestation.

When evaluating neurodevelopmental outcomes utilizing the split-week gestation model, statistically significant differences were noted only in infants between the early and late split of the gestational week at 23 weeks (early vs. late), 13.3% vs. 54.5% for no neurodevelopmental impairment, 33.3% vs. 22.7% for moderate impairment, and 53.3% vs. 22.7% for significant impairment (*p* = 0.034), respectively. There were no differences seen in the split-week model for 24, 25, and 26 weeks.

## 4. Discussion

The objective of this study was to compare the neurodevelopmental outcomes of a surviving cohort of infants born between 23 and 26 weeks gestation and discharged home, using a split-week gestational model (early versus late) GA and the standard completed GA categorization. In this large cohort of extremely preterm infants, we found that there were no differences seen in the split-week model for 24, 25, and 26 weeks gestation. There was a difference seen in the split-week observations at 23 weeks gestation, with the early part faring more poorly compared to the latter part. This contrasts with the findings seen in the earlier study from the same cohort, which showed a significant difference in the composite outcome of neonatal mortality or morbidity between the early and late split of a gestational week at 24, 25, and 26 weeks, but not at 23 weeks. Likely, the sample size at 23 weeks gestation in this cohort was not adequate to reveal a real difference in contrast to work by Nguyen et al., showing a statistically significant difference even at 23 weeks gestation. The finding of a statistically significant difference in the long-term outcomes at 23 weeks gestation as compared to the more mature infants using the split-week gestational model may be a reflection of specific neonatal care challenges in supporting maturation through optimal nutrition while minimizing iatrogenic injury to the more immature infant.

There have been several recent studies evaluating the neurodevelopmental outcomes of extremely preterm infants during similar periods as the cohort included in this study. It was reassuring that the neurodevelopmental outcomes of this cohort were comparable or better to some of these recent studies [1,2,3,4,5,6]. However, many of the studies grouped their gestational age categories, so direct comparisons may be challenging, but the trend was similar. In the EPIPAGE-2 cohort study, there was only one survivor at 23 weeks and outcomes were available for 24, 25, and 26 weeks; the incidence for no impairment for 24 weeks was 29.3% (22.6–35.9%), for 25 weeks was 54.6% (48.8–60.3%), and for 26 weeks 69.8% (65.2–74.3%) [1]. In a centre that practices active management for infants at the limits of viability, no or mild neurodevelopmental impairment in surviving infants at 22–23 weeks (*n* = 70) were 64% and 76% for infants at 24–25 weeks (*n* = 178) [6].

Where does and can the split-week GA model approach assist in providing needed additional information when counseling parents? Guidelines are clear that counseling at the limits of viability, namely 23 and 24 weeks, should and is a shared decision-making process based on ensuring that all the known information can be provided to the parents [16]. The split-week GA model results reinforced that neurodevelopmental outcomes in extreme preterm infants between 24 and 26 weeks gestation are not as affected on a day-by-day basis as compared to mortality and neonatal outcomes. Parents will need to understand this concept in their decision-making process, especially at the extremes of viability. If antenatal management is successful in continuing a pregnancy for several more days, particularly to allow for antenatal corticosteroids to direct their effects on the fetal lungs and stabilize the transition to ex utero life for the preterm infant, it can provide some direction as to how a preterm infant will adjust once it is born. However, the model provided different information for the less mature infant at 23 weeks gestation. Here, the model demonstrated that the latter half of the week proved to be more beneficial from a neurodevelopmental perspective, but only if the infant survives. The number of 23-week infants in this cohort was small for the study period, but the differences in the split model observations were significant, suggesting that this was a real finding. The earlier part of 23 weeks gestation may represent a degree of immaturity and consequent morbidity from which there is a greater risk of long-term neurodevelopmental impairment in survivors. Future studies of a larger number of babies born at 22 and 23 weeks gestation may provide greater insight into this finding.

Research has shown that parents have expressed the need for clear, consistent counseling with more interpretable information, especially in the event of an extreme preterm birth and subsequent care [17,18]. The need to add as many days as possible for the vulnerable fetus plays a role in reducing both mortality and significant neonatal outcomes. This was noted in the earlier study mentioned herein. The reduction of serious neonatal outcomes, namely chronic lung disease, severe retinopathy of prematurity, and brain injury, may then play a significant role in the gradual improvement of neurodevelopmental outcome measures [8,19]. In addition, one may need to reconsider what components of the commonly reported currently established neurodevelopmental outcomes should be included in antenatal counseling, and if others should now also be considered; parental input and feedback should be part of that process and the split-week model revisited those measures [17].

### 4.1. Strengths

The data were derived from a large cohort of infants admitted to two similar tertiary-care perinatal centres in Canada over the same 10-year period. Both perinatal centres followed a similar approach in the neurodevelopmental monitoring of the survivors from their respective centres, thus ensuring the reasonable quality of the data.

### 4.2. Limitations

There are several limitations to our study. Despite the large sample size, there was a small number of infants included at 23 weeks gestation. Although we are confident with the findings for this gestation, studies with a larger sample size and the inclusion of infants born at 22 weeks should be conducted to validate this observation. A second limitation is that we did not collect the age at which some of the components of the follow-up information were collected, in particular, the age at which the BSID-III was completed.

## 5. Conclusions

In contrast to our earlier study, the split-week model did not provide additional information for pregnancies and infants between 24 and 26 weeks gestation. It did, however, provide information for counsel for infants at 23 weeks gestation, which showed benefits in the late half versus the early half of the week. This information should be combined with a body of information that a clinician can utilize in their counseling sessions with parents in the face of extreme preterm birth.

## Figures and Tables

**Table 1:** Categorization of neurodevelopmental outcome measures by 18–24 months corrected age.

Diagnostic Criteria	Normal or Mild Impairment	Moderate Impairment	Severe Impairment
Cognitive	BSID-III cognitive composite score >85	BSID-III cognitive composite score 70–85	BSID-III cognitive composite score <70
Motor	BSID-III motor composite score >85 or No diagnosis of CP or mild CP (GMFCS 0, 1)	BSID-III motor composite score 70–85 or Diagnosis of CP with GMFCS level 2 (walks with orthotics)	BSID-III motor composite score <70 or Diagnosis of CP with GMFCS level 3–5 (walks using a hand-held mobility device or self-mobility with a powered mobility device or transported in a manual wheelchair)
Language	BSID-III language composite score ≥85	BSID-III language composite score 70–84	BSID-III language composite score <70
Vision	Mild visual impairment (visual acuity better than 20/200 in both eyes)	Bilateral blindness (visual acuity less than 20–200 in the strongest eye)	Bilateral blindness that cannot be corrected
Hearing	Mild hearing impairment (not requiring amplification or requiring amplification in just one ear)	Bilateral hearing loss (requiring amplification)	Severe to profound hearing impairment (no functional hearing with amplification)

**Table 2:** Characteristics (maternal, neonatal) among all survivors (followed and not followed).

	Gestational Weeks			
**Maternal Characteristics and Morbidity among Mothers for Surviving Infants**	**23 Weeks** **(*n* = 48)**	**24 Weeks** **(*n* = 246)**	**25 Weeks** **(*n* = 383)**	**26 Weeks** **(*n* = 433)**
Maternal age, mean (SD)	29.94 (5.46)	30.62 (5.51)	30.68 (6.07)	30.46 6.08)
Gravidity, mean (SD)	2.79 (1.95)	2.52 (1.66)	2.61 (1.92)	2.49 (1.72)
Maternal Education, *n* (%)				
Less than grade 12 equivalent	9 (20.0)	23 (10.6)	27 (7.8)	37 (9.9)
High school graduate	13 (28.9)	53 (24.4)	66 (19.2)	91 (24.3)
Some post-secondary degree	12 (26.7)	41 (18.9)	66 (19.2)	78 (20.8)
University/graduate degree	11 (24.4)	100 (46.1)	185 (53.8)	169 (45.1)
Unknown	3 (6.0)	29 (12.0)	39 (10.0)	58 (13.0)
Single parent family, *n* (%)	6 (13.6)	20 (9.4)	32 (9.3)	35 (9.4)
Obesity (weight > 91 kg), *n* (%)	0 (0.0)	10 (4.1)	9 (2.4)	15 (3.5)
Pre-pregnancy hypertension, *n* (%)	0 (0.0)	2 (0.8)	10 (2.6)	14 (3.2)
PIH, Gestational hypertension, Pre-eclampsia, *n* (%)	0 (0.0)	8 (3.3)	27 (7.1)	57 (13.2)
Assisted conception, *n* (%)	8 (17.0)	53 (21.7)	55 (14.4)	68 (15.7)
Smoking during pregnancy, *n* (%)	7 (14.6)	25 (10.2)	43 (11.3)	54 (12.5)
Alcohol use during pregnancy, *n* (%)	1 (2.1)	13 (5.3)	12 (3.1)	11 (2.5)
Multiple births, *n* (%)	13 (27.1)	66 (26.9)	97 (25.3)	112 (25.9)
Antenatal corticosteroids, *n* (%)	36 (76.6)	222 (90.2)	353 (92.4)	391 (90.9)
PPROM > 24 h, *n* (%)	16 (34.0)	61 (24.9)	110 (28.9)	117 (27.0)
Chorioamnionitis, *n* (%)	15 (31.2)	84 (34.1)	130 (34.1)	114 (26.3)
Antepartum hemorrhage, *n* (%)	14 (29.2)	74 (30.1)	93 (24.5)	116 (26.8)
Outborn, *n* (%)	14 (29.2)	45 (18.3)	64 (16.70	89 (20.6)
Caesarean birth, *n* (%)	11 (22.9)	126 (51.2)	204 (53.3)	256 (59.1)
**Neonatal Characteristics and Morbidity of Surviving Infants**	**23 Weeks** **(*n* = 48)**	**24 Weeks** **(*n* = 246)**	**25 Weeks** **(*n* = 383)**	**26 Weeks** **(*n* = 433)**
Birth weight, mean (SD)	591.77 (65.14)	677.00 (90.22)	766.97 (104.00)	866.25 (152.73)
Small for gestational age, *n* (%)	3 (6.2)	14 (5.7)	13 (3.4	43 9.9
Female, *n* (%)	23 (47.9)	122 (49.6)	202 (52.7)	203 (46.9)
Necrotizing enterocolitis (≥Bell stage 2), *n* (%)	7 (14.6)	28 (11.4)	48 (12.5)	37 (8.5)
Retinopathy of prematurity, *n* (%)				
Stage 3	28 (58.3)	92 (37.7)	75 (19.6)	40 (9.3)
Stage 4, 5	0 (0)	1 (0.4)	1 (0.3)	0 (0)
Sepsis (culture-proven), *n* (%)	26 (54.2)	96 (39.3)	113 (29.6)	102 (23.6)
Intraventricular hemorrhage, *n* (%)				
Grade 1	13 (27.1)	54 (22.0)	77 (20.1)	70 (16.2)
Grade 2	6 (12.5)	29 (11.8)	47 (12.3)	29 (6.7)
Grade 3	4 (8.3)	11 (4.5)	16 (4.2)	16 (3.7)
Grade 4/periventricular venous infarct	7 (14.6)	32 (13.1)	34 (8.9)	21 (4.8)
Periventricular leukomalacia, *n* (%)	2 (4.2)	5 (2.0)	17 (4.4)	14 (3.2)
Hypotension requiring inotropes, *n* (%)	31 (64.6)	87 (35.5)	92 (24.0)	82 (18.9)
Bronchopulmonary dysplasia, *n* (%)	38 (79.2)	158 (65.0)	183 (48.3)	154 (36.7)
Days of respiratory support, median (IQR)	78.50 (62.00, 89.50)	66.00 (54.00, 84.00)	56.00 (43.00, 69.00)	43.00 (32.00, 55.00
Postnatal corticosteroids, *n* (%)	17 (35.4)	58 (23.6)	53 (13.8)	30 (6.9)
Length of stay (days) for initial hospitalization, median (IQR)	127.00 (116.50, 139.50)	118.00 (106.00, 134.00)	104.00 (91.00, 123.00)	90.00 (78.00, 103.00)

SD = standard deviation, IQR = interquartile percentiles.

**Table 3:** Neurodevelopmental characteristics at 18–24 months corrected age of infants who were followed.

	Gestational Weeks			
**Neurodevelopmental Characteristics**	**23 Weeks** **(*n* = 45)**	**24 Weeks** **(*n* = 224)**	**25 Weeks** **(*n* = 360)**	**26 Weeks** **(*n* = 383)**
**Growth**				
Head circumference, mean (SD)	46.26 (1.67)	46.53 (1.70)	46.93 (1.82)	47.24 (0.72)
Weight, mean (SD)	10.17 (1.50)	10.19 (1.52)	10.56 (1.52)	10.76 (1.57)
Height, mean (SD)	80.19 (3.77)	80.07 (3.40)	80.27 (2.99)	80.84 (3.46)
Post-discharge morbidity, *n* (%)				
Discharged home on oxygen	26 (57.8)	92 (41.1)	87 (24.2)	77 (20.1)
Gastrostomy tube feedings	7 (15.9)	18 (8.1)	18 (5.0)	12 (3.1)
Hospitalization	18 (40.0)	90 (40.2)	126 (35.0)	103 (27.0)
Need for recurrent medications	16 (37.2)	64 (29.1)	84 (23.7)	74 (19.7)
Seizure disorder	1 (2.4)	7 (3.2)	8 (2.3)	2 (0.5)
Ventriculoperitoneal shunt	1 (2.2)	11 (4.9)	13 (3.6)	6 (1.6)
**BSID-III Measures**				
BSID-III completion, *n* (%)	33 (73.3)	175 (78.10)	303 (84.2)	330 (86.2)
Cognition composite score, mean (SD)	85.38 (15.06)	88.63 (14.45)	92.51 (13.95)	94.08 (12.35)
median (IQR)	90.00 (80.00, 95.00)	90.00 (80.00, 100.00)	95.00 (85.00, 100.00)	95.00 (85.00, 105.00)
Language composite score, mean (SD)	77.43 (16.16)	83.09 (16.97)	87.72 (15.46)	87.79 (14.99)
median (IQR)	74.00 (66.50, 92.50)	84.50 (71.00, 94.00)	89.00 (77.00, 100.00)	89.00 (77.00, 100.00)
Motor composite score, mean (SD)	82.69 (16.75)	88.50 (14.13)	91.68 (13.15)	92.77 (12.41)
median (IQR)	88.00 (70.00, 95.50)	94.00 (82.00, 97.00)	94.00 (85.00, 100.00)	94.00 (85.00, 100.00)
**Neurosensory/Neuromotor**				
Hearing Impairment, *n* (%)				
Mild	2 (4.4)	13 (5.8)	8 (2.2)	5 (1.3)
Severe	1 (2.2)	14 (6.3)	14 (3.9)	8 (2.1)
Vision Impairment, *n* (%)				
Mild	15 (33.33)	36 (16.1)	19 (5.3)	16 (4.2)
Severe	1 (2.2)	6 (2.7)	9 (2.5)	5 (1.3)
Motor Impairment (Cerebral palsy), *n* (%)				
None	39 (86.7)	190 (87.6)	318 (90.3)	356 (93.9)
Mild (GMFCS 1, 2)	2 (4.4)	8 (3.7)	23 (6.6)	15 (4.0)
Moderate/severe (GMFCS 3, 4, 5)	4 (8.8)	19 (9.13)	11 (3.1)	8 (2.2)

SD = standard deviation, IQR = interquartile percentiles, BSID = Bayley Scales of Infant Development, GMFCS = Gross Motor Functional Classification System.

**Table 4:** Primary outcomes.

Gestational Age (Weeks)		Outcome *n* (%)			*p*-Values
		No Impairment	NDI ^β^	SNI *	
23	Full Early split Late split	14 (37.8) 2 (13.3) 12 (54.5)	10 (27.0) 5 (33.3) 5 (22.7)	13 (35.1) 8 (53.3) 5 (22.7)	0.034
24	Full Early split Late split	94 (49.0) 51 (45.9) 43 (53.1)	55 (28.6) 34 (30.6) 21 (25.9)	43 (22.4) 26 (23.4) 17 (21.0)	0.615
25	Full Early split Late split	202 (65.0) 111 (63.8) 91 (66.4)	66 (21.2) 39 (22.4) 27 (19.7)	43 (13.8) 24 (13.8) 19 (13.9)	0.841
26	Full Early split Late split	227 (69.0) 127 (65.1) 100 (74.6)	77 (23.4) 53 (27.2) 24 (17.9)	25 (7.6) 15 (7.7) 10 (7.5)	0.138

* Significant neurodevelopmental impairment: composite of at least one of BSID-III mean cognitive, language, or motor score <70; cerebral palsy with GMFCS score of 3, 4, or 5; severe hearing impairment; severe visual impairment. ^β^ Neurodevelopmental impairment: composite of at least one of BSID-III mean cognitive, language, or motor score 70–84; cerebral palsy with a GMFCS score of 1 or 2; mild hearing impairment; mild visual impairment.

## Data Availability

Data supporting this project can be found at the Department of Pediatrics, Cumming School of Medicine, University of Calgary, Calgary, AB, Canada and are available on request to Sumesh Thomas.
